# Impact of gist intervention on automated system interpretability and user decision making

**DOI:** 10.1186/s41235-024-00594-2

**Published:** 2024-10-09

**Authors:** Lydia P. Gleaves, David A. Broniatowski

**Affiliations:** Department of Engineering Management and Systems Engineering, The George Washington University, 800 22nd St. NW, Washington, DC 20052 USA

**Keywords:** Fuzzy-trace theory, Gist, Individual differences, Explainability, Interpretability, Misinformation

## Abstract

As they become more common, automated systems are also becoming increasingly opaque, challenging their users’ abilities to explain and interpret their outputs. In this study, we test the predictions of fuzzy-trace theory—a leading theory of how people interpret quantitative information—on user decision making after interacting with an online decision aid. We recruited a sample of 205 online crowdworkers and asked them to use a system that was designed to detect URLs that were part of coordinated misinformation campaigns. We examined how user endorsements of system interpretability covaried with performance on this coordinated misinformation detection task and found that subjects who endorsed system interpretability displayed enhanced discernment. This interpretability was, in turn, associated with both objective mathematical ability and mathematical self-confidence. Beyond these individual differences, we evaluated the impact of a theoretically motivated intervention that was designed to promote sensemaking of system output. Participants provided with a “gist” version of system output, expressing the bottom-line meaning of that output, were better able to identify URLs that might have been part of a coordinated misinformation campaign, compared to users given the same information presented as verbatim quantitative metrics. This work highlights the importance of enabling users to grasp the essential, gist meaning of the information they receive from automated systems, which benefits users regardless of individual differences.

## Introduction

With the proliferation of automated decision aids, such as machine learning applications, into workplaces and everyday life, more fields than ever are relying on the output and decisions of these tools (Lai et al., 2021). Their complexity has made it increasingly difficult for most users to understand their output. Predictions are becoming increasingly accurate through the use of “black box” models, such as deep neural networks; however, these complex models often lack transparency compared to simpler models with fewer parameters (Guidotti et al., 2018; Heinrichs and Eickhoff, 2020). Nevertheless, these opaque algorithms are increasingly used to make consequential societal and personal decisions.

Users given interpreted information (not just literal facts) are likely to make better or more correct decisions, and users are more likely to take a system’s output information into account when they understand it. Because of this, interpretability and explainability are currently generally recognized as important requirements for machine learning systems (Doshi-Velez and Kim, 2017). There is a widespread notion that increased use of techniques that inform users of how systems work will lead to improved comprehension and trustworthiness (Adadi and Berrada, 2018). However, to date there is limited empirical evidence to support this assertion.

In this study, we draw on fuzzy-trace theory, an empirically validated account of how humans make decisions based on their interpretations of numerical, verbal, and pictorial stimuli, to make predictions about how perceived and actual model utility varies among different users (Reyna, 2012). According to fuzzy-trace theory, model output is encoded into human memory as several simultaneous mental representations that vary in precision. The most precise of these representations is referred to as the verbatim representation.

For example, during the height of the COVID-19 pandemic, verbatim URL credibility statistics (typically presented on a scale of 0–100) were widely used to inform users about the potential for a news article to contain misinformation. Prior work (Broniatowski et al., 2022) found that content from “Intermediate Credibility” URLs, which often had ratings that were high enough to be plausible (e.g., because they had the trappings of legitimate news sources), but nevertheless occasionally distorted the truth, were among those most likely to go viral. Verbatim ratings for these sources was often above 50 on a scale of 0–100, which might have led users to conclude that they were “mostly credible” and should therefore be believed. Although prior work shows that credibility ratings can facilitate misinformation discernment (Pennycook and Rand, 2019a), fuzzy-trace theory suggests that communicating the gist that these sources might have been “possibly not credible” may have made them even less compelling (see discussion of possibility vs probability in (Reyna et al., 2022)). Similarly an automated tool may be used to generate quantitative scores that help system users make judgments about whether information that is shared online comes from a trustworthy source. The tool may report that 52.37% of the shares associated with a particular news article come from the 10% most active accounts. The verbatim representation of this statistic would be a precise recapitulation of the stimulus—subjects who rely upon verbatim memory would be expected to answer “52.37%” when asked how many shares associated with the news article come from the 10% most active accounts. However, this number may be difficult to interpret without context such as what proportion of shares typically are generated by the 10% most active accounts. In this case, such a large concentration of shares from a small number of users is substantially higher than what one might normally expect and may therefore be one indication of a coordinated influence campaign (Giglietto et al., 2020). Thus, in contrast to the decontextualized, precise, verbatim representation, subjects relying upon imprecise, yet meaningfully interpreted, *gist* representations might respond that the proportion of shares coming from the 10% most active users is “suspiciously high”. Therefore, in this study, we examine whether subjects using a similar tool can identify indicators of coordinated misinformation campaigns beyond the effects of providing verbatim statistics.

Humans may encode several gist representations of a model’s output which differ in their levels of precision and reliance on context. For example, if a model is going to be used to make a decision, fuzzy-trace theory predicts that people make decisions based on the simplest gist interpretation that helps subjects distinguish between options in context. Consequently, models are likely to be considered interpretable if they communicate the gist to subjects in a manner that helps them to make decisions (Broniatowski, 2021).

### Mental representations and improved judgment

According to Reyna and Brainerd (2023), a literal focus on objective, verbatim data alone does not empower users to understand the meaning of system output. To better enable this understanding, users should be provided the gist—or empowered with the ability to extract the gist—of the output. To accomplish this, information should be organized in a meaningful and interpretable way. Studies designed with fuzzy-trace theory in mind have demonstrated that emphasizing the gist of a system output or idea, not just the verbatim representation, inspires greater confidence, trust, and understanding in its users. For example, Cozmuta et al. (2018) found that icon arrays were most effective for increasing patients’ likelihoods of taking a new medication when they contained information explaining the gist of the risks and benefit, especially among patients with low numeracy. Our work builds upon these insights and prior applications of fuzzy-trace theory to automated decision support (Wolfe et al., 2013) by investigating whether a gist-based tutorial will improve users’ decision making.

### Relationship to other factors theorized to improve judgments

Prior work has associated several other factors with improved use of automated decision aids.

#### Work experience

Experienced workers typically outperform novices (Ericsson et al., 2018). Although this is due, in part, to experts’ increased knowledge and training, fuzzy trace theory moves beyond these factors in positing an increased reliance on gist mental representations. Gist is developmentally advanced in that experts, more so than novices, tend to rely on gist representations when deciding (Reyna, 2018; Reyna et al., 2014). Thus, fuzzy-trace theory predicts a role for gist that moves beyond the additional knowledge often possessed by experts.

#### Cognitive reflection

Another body of work is based on standard dual process theories—which posit that decision making is driven by a combination of intuitive and non-rational mental processes, on the one hand, and rational, reflective processes, on the other hand. This work suggests that subjects who are low in cognitive reflection—i.e., those who are unable to suppress intuitive, yet incorrect, responses—are less likely to accurately detect misinformation (Frederick, 2005; Pennycook, 2023; Pennycook and Rand, 2019b; Thomson and Oppenheimer, 2016). Literature supporting the role of cognitive reflection builds upon a tradition growing out of the work of Tversky and Kahneman (Tversky and Kahneman, 1974; Tversky et al., 1982) who argue that people rely on heuristics and biases when making decisions and judgments. Standard dual process thinking posits that fast thinking is cognitively less advanced (compared to reflective thinking). Fuzzy-trace theory moves beyond these standard dual process theories by positing that gist-based intuitive thinking is cognitively more advanced (compared to literal verbatim processing) (Reyna, 2018; Reyna et al., 2021).

#### Numeracy

Decision makers who are more *numerate* tend to have better decision outcomes when faced with mathematical tasks such as those requiring interpretation of numerical data generated by automated tools. Furthermore, objective mathematical ability and mathematical self-confidence (subjective numeracy, which may be further subdivided into assessments of one own’s mathematical ability and preference to rely on mathematics) both contribute unique sources of variance to decision quality in both medical and financial settings (Fagerlin et al., 2007; Liberali et al., 2012; Peters et al., 2019).

Beyond the role of numeracy, other scholars have also examined the role of meaning in improving numerical decision making. Hibbard and Peters (2003) found that highlighting the meaning of information when presenting it enabled less numerate people to have higher comprehension and make better decisions than when presented with the information in a more verbatim-type way. Although Hibbard and Peters (2003) explained their findings as a consequence of a reduced cognitive load, gist is crucially not the same thing as a reduction in cognitive load (for critical tests, see Reyna and Brainerd (1995)).

### Hypotheses

The above discussion motivates the key hypothesis of this work: subjects presented with the gist of an automated system’s output will make better (i.e., more correct) judgments than those given only the same information presented in a verbatim manner. Furthermore, we examine whether gist adds explanatory power beyond other factors that would be expected to improve decision outcomes based on the literature discussed above. Specifically, we examine whether gist explains significant variance beyond the effects of self-reported measures of expertise, cognitive reflection, and numeracy (both objective and subjective). Since people may not adopt systems if they feel that they do not find them useful, we also examined factors that might explain subjects’ perceptions of system utility, such as interpretability and explainability.

We test this hypothesis using an online decision aid system called “Information Tracer” (Chen et al., 2021). Information Tracer is designed to respond to a pervasive challenge of misinformation across various domains, including politics and public health. This misinformation is often spread using automated software to promote its visibility on social media platforms by creating the false impression of widespread consensus (Ayers et al., 2021; Broniatowski et al., 2018; Giglietto et al., 2020). The ability to discern trustworthy information is crucial for the average consumer of information, yet the “ground truth” regarding a message’s origin (coordinated misinformation campaign or not) is often difficult to ascertain. Information Tracer is therefore intended to help users evaluate the credibility and potential bias of information they encounter online. It focuses specifically on identifying coordinated misinformation campaigns, where misleading information is spread in a planned and coordinated manner. Information Tracer utilizes various techniques, including analyzing text data, to identify patterns indicative of such campaigns.

## Method

### Sample

This experiment was performed using a sample of online microworkers from Amazon’s Mechanical Turk (MTurk) platform between July 18 and August 1, 2021. Of 240 MTurk users who completed the survey, 17 (7%) failed one or more attention check questions and were excluded from further analysis. Of the remaining 223 subjects, 18 (8%) did not complete the Information Tracer tutorial and were therefore also excluded. Of the remaining 205 participants, 117 (57%) were men (87 women, 1 other). 165 (81%) were white, and 156 (76%) held at least a four-year college degree. Further demographic data are available in Table 1.

**Table 1:** Sociodemographic characteristics of participants recruited from MTurk

		*n*	%
Gender			
	Male	117	57.1
	Female	87	42.4
	Other	1	0.5
Race			
	Asian	12	5.6
	Black/African American	23	11.2
	Native American/American Indian/Alaskan Native	2	1.0
	White	165	80.5
	Other	3	1.5
Ethnicity			
	Hispanic	18	8.8
	Non-Hispanic	187	91.2
Highest educational level			
	Obtained a graduate/professional degree	28	13.7
	Graduated from a 4-year college or more	128	62.4
	Attended some college but did not finish a 4-year degree	35	17.1
	Graduated from high school	14	6.8

*N* = 205. Participants were on average 37.5 years old (SD = 10.2), and participant age did not differ significantly by condition. The full demographic questionnaire is shown in Appendix F

### Instruments

#### URL coordination judgments

Our primary measures were intended to index subjects’ ratings of whether a Uniform Resource Locator (URL; i.e., weblink) shown by Information Tracer was part of a coordinated misinformation campaign.

**Fig. 1:**
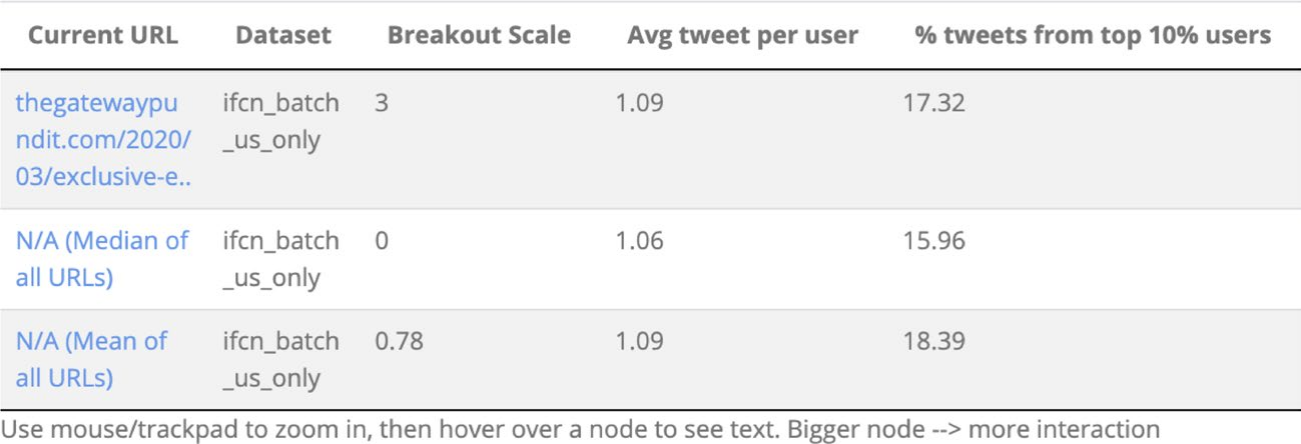
URL statistics as provided to users by information tracer

*Verbatim metrics* For each URL, subjects were asked to report the exact values of ten outputs produced by Information Tracer and presented to users, such as those shown in Fig. 1. The full set of outputs elicited may be found in Appendix A. Three of these outputs correspond to metrics that are diagnostic of coordinated misinformation campaigns based on prior literature. These were:

- The platform reach, or “breakout scale” (how many social media platforms, such as Facebook, Twitter, and Reddit, the URL had spread to). The breakout scale reflects the fact that coordinated disinformation campaigns frequently attempt to spread the same content across multiple social media platforms simultaneously (Nimmo, 2020).
- The average number of tweets per user who shared the URL on Twitter
- The proportion of tweets containing the URL that came from the top 10% of users (how top-loaded the discussion was).

These latter two metrics capture the extent to which a small number of accounts may be artificially amplifying access to online content, and are thus intended to measure whether traffic on social media platforms is being artificially manipulated. These metrics were developed by the Information Tracer team based upon patterns observed in prior documented online information operations (Chen et al., 2021; Nimmo, 2019 ). We therefore asked subjects to input the exact values of these three metrics for each URL shown to participants. We next applied logarithmic transforms to the breakout scale and average number of tweets per user metrics, since subjects’ responses to these were highly skewed. Subjects’ responses to these three measures nevertheless were not internally consistent, *Cronbach’s *$\alpha = 0.19$. For each metric, we therefore recorded whether it was reported correctly (1) or incorrectly (0) with item responses were averaged into a common scale. This correctness scale was highly reliable, *Cronbach’s *$\alpha = 0.94$.

*Gist judgments* Subjects were also asked to answer eight Likert-scale items indexing their rating of the URL as part of a coordinated misinformation campaign. All items in this section were assessed on a 7-point Likert scale ranging from strongly disagree (− 3) to strongly agree (+ 3). The specific items were (items marked as “R” were reverse-coded):

- *This URL shows evidence of coordinated posting*
- *This URL is part of a coordinated misinformation campaign*
- *This URL is primarily being spread by automated accounts (“bots”)*
- *This URL is NOT primarily being spread organically (by human users)*
- *This URL is primarily being spread organically (by human users) (R)*
- *This URL does NOT show evidence of coordinated posting (R)*
- *This URL is NOT part of a coordinated misinformation campaign (R)*
- *This URL is NOT primarily being spread by automated accounts (“bots”) (R)*

These items were reliable, *Cronbach’s* $\alpha = 0.80$ and were therefore averaged into a single 7-point Likert scale.

#### System utility evaluation

Subjects who completed the Information Tracer task were asked to complete several system evaluation items that were adapted from those used in a pilot study(Gleaves et al., 2020). This pilot study yielded four distinct factors: whether a participant was able to discover new things (Discovery), to explain how system output was generated (Explainability), to make sense of system output (Interpretability), and to make use of data visualizations (Visualization). We retained the three pairs of items per factor that resulted in the highest reliability (as measured by Cronbach’s $\alpha$) in this pilot study, for a total of 18 items as follows:

*Interpretability scale* These items indexed whether participants could make sense of system output as follows (items marked as “R” were reverse-coded):

- *I can explain what the system’s results mean*
- *I can make sense of what the system’s results are saying*
- *The system’s results make sense to me*
- *I CANNOT explain what the system’s results mean (R)*
- *I CANNOT make sense of what the system’s results are saying (R)*
- *The system’s results do NOT make sense to me (R)*

These items were highly reliable, *Cronbach’s *$\alpha$ *= 0.91*.

*Explainability scale* These items indexed whether participants could explain how system output was generated as follows (items marked as “R” were reverse-coded):

- *I can explain how the system generated its output*
- *The system allowed me to see how it generated its output*
- *The system explained to me how it generated its output*
- *I CANNOT explain how the system generated its output (R)*
- *The system did NOT allow me to see how it generated its output (R)*
- *The system did NOT explain to me how it generated its output (R)*

These items were highly reliable, *Cronbach’s* $\alpha$ *=0.89*

*Discovery scale* These items indexed whether participants could use the system to discover new insights as follows (items marked as “R” were reverse-coded):

- *The system provided me with new insight*
- *The system helped me to think of something new*
- *The system helped provide a fresh perspective*
- *The system did NOT provide me with new insight (R)*
- *The system did NOT help me to think of something new (R)*
- *The system did NOT help provide a fresh perspective (R)*

These items were highly reliable, *Cronbach’s* $\alpha = 0.89$

*Factual accuracy judgments* In addition to the items in the interpretability, explainability, and discovery subscales, we also included two items indexing whether the system helped users to make factual accuracy judgments as follows.

- *The system helped me assess whether the websites were factually accurate*
- *The system did NOT help me assess whether the websites were factually accurate (R)*

These items were significantly anticorrelated $r(203)=-0.52,\,{p}\,<\,0.001$ and were therefore averaged after reversing the sign of the second item. All items in the system utility evaluation were assessed on a 9-point Likert scale ranging from strongly disagree (− 4) to strongly agree (+ 4). Free-response items indexing additional perceptions of system utility were also included (see Appendix B).

#### Individual difference measures

All subjects were presented with a composite questionnaire including published measures of individual skills and traits that we expected to be associated with performance on the Information Tracer task. These measures included scales designed to assess self-reported measure of expertise, cognitive reflection, and numeracy. Specifically, we included a widely used scale of objective numeracy (Lipkus et al., 2001), the subjective numeracy scale, which we divided into subscales indexing mathematical ability and preference for numerical information (Fagerlin et al., 2007) and all items from the first (Frederick, 2005) and second (Thomson and Oppenheimer, 2016) Cognitive Reflection Tests, which we combined as in prior work (Pennycook and Rand, 2019b). The number of items and reliability values for these scales are shown Table 2.

**Table 2:** Descriptions of instruments used to measure individual differences

Instrument		Items	Scoring	$\alpha$	Reference
Cognitive reflection test		7	% Correct	0.82	
	CRT-1	3		0.79	Frederick (2005)
	CRT-2	4		0.65	Thomson and Oppenheimer (2016)
Objective numeracy		11	% Correct	0.89	Lipkus et al. (2001)
Subjective numeracy		7	6-point Likert	0.84	Fagerlin et al. (2007)
	Ability subscale	3		0.82	
	Preference subscale	4		0.81	

Responses for items in the combined cognitive reflection test questionnaires and expanded numeracy scale were scored by the percentage of the participant’s correct responses. The subjective numeracy scale was scored as instructed in its source material by reverse-coding negatively framed items before averaging Likert responses. One item indexing subjective mathematical ability—“How good are you at figuring out how much a shirt will cost if it is 25% off?”—was inadvertently not included in the subjective numeracy scale

#### Self-reported work experience

We collected participants’ ratings of their own data science expertise using the following items:

- *Do you currently use a formal method to carry out your work? (Yes/No)*
- *Do you currently use machine learning or other Artificial Intelligence (AI) techniques to carry out your work? (Yes/No)*
- *Please select your data science experience from the list below (this item was coded on a 5-point Likert scale as the highest box checked):*
  1. *I have no experience in data analysis*
  2. *I have no coursework or professional experience with data modeling*
  3. *I have experience in data analysis*
  4. *I have some coursework in data modeling and/or engineering*
  5. *I have extensive coursework and professional experience in data modeling and/or engineering*

These items were then combined into a 7-point scale by adding the number of “yes” responses from the first two items to the Likert-scale value of the last item. All scales were administered using Qualtrics survey software, with both the order of instruments and items within these instruments randomized.

### Materials

#### Tutorials

*Verbatim* All participants received a link to a detailed verbatim description of Information Tracer, which included a video tutorial describing the system. The verbatim description was based on a tutorial written by the system’s designers (Chen et al., 2021) and described the mechanics of the system and how measures were calculated. For example, when describing the breakout scale metric, the verbatim tutorial described it as:

*Breakout scale* The number of other social media platforms with links pointing to the original post containing this URL.

- *A URL is said to “break out” on a platform if there are more than 100 interactions with the URL*
- *For example, if an article was only shared on Twitter and has fewer than 100 retweets and/or replies, the breakout scale is 0.*
- *If an article is widely shared on Twitter (100+ retweets and/or replies), and there are fewer than 100 posts linking to these tweets on other platforms, the breakout scale is 1.*
- *If the article was widely shared on Twitter and Facebook and was once posted on Reddit but had zero comments, then the breakout scale is 2.*

In addition, the breakout scale metric for this URL shown in Fig. 1 was outlined with a red box.

This description guided users through the use of the Information Tracer tool, beginning with choosing a URL to assess. It provided detailed information about how the breakout scale, average number of tweets per user, and percentage of tweets from the top ten percent of users were calculated, but did not communicate why these metrics were chosen or how to interpret them. It also provided detailed information about the tables and figures that allowed users to explore social media data, including viewing particular posts. Thus, the verbatim “tutorial” only provided information describing the system’s outputs, without interpreting them; no guidance was provided to participants regarding how to make a judgment about whether a URL is part of a coordinated misinformation campaign. The full verbatim description is available in Appendix C.

*Verbatim + Gist* Those in the verbatim + gist condition received an additional gist-based explanation of the system after the verbatim tutorial. This gist-based explanation, titled “The Bottom Line,” focused on interpreting what the metrics mean regarding whether a URL was spread as part of a coordinated misinformation campaign. For example, when describing the breakout scale metric, the gist tutorial stated:

*If the URL has a high breakout score, that means it got many comments, replies, or retweets on several social media platforms. A high score might indicate a coordinated misinformation campaign because the more platforms an article is shared on, the higher the audience of users that can be targeted and manipulated. Most URLs have a breakout score of 1 or lower.*

As in the verbatim tutorial, the breakout scale metric for this URL shown in Fig. 1 was outlined with a red box. However, beyond telling participants how the numbers were calculated, this tutorial emphasized the meaning of the numbers in the context of deciding whether URLs were part of a coordinated misinformation campaign. The full gist tutorial is available in Appendix D.

Importantly, each tutorial version lies on a spectrum. The more “verbatim” content provides more highly specific information and is on the more verbatim end of the theoretical continuum while the more “gist” content focuses on core concepts and helps people to contextualize the system’s output and to make sense of it.

#### URLs

Participants used the Information Tracer system to track the spread of one of three URLs over multiple social media platforms and to decide whether it was part of a possible coordinated misinformation campaigns based on a set of metrics. These statistics, and corresponding baseline values, were presented to users in the format shown in Fig. 1. The three URLs used, and their classifications according to the statistics provided by Information Tracer, are available in Table 3. Each URL pointed to a news article if users chose to follow it. Each article contained COVID-19 misinformation, with topics ranging from religious perspectives to international news.

**Table 3:** URL coordination statistics

	Definitely coordinated	Possibly coordinated	Not coordinated
Breakout score	2	3	1
Average number of tweets per user	1.11	1.08	1.00
Percent of tweets from top 10% of users	19.51	16.78	11.11

Definitely coordinated URL: http://fromrome.info/2020/04/05/bill-gates-my-corona-stunt-requires-18-months-of-control-then-mandatory-vaccination Possibly coordinated URL: https://www.zerohedge.com/geopolitical/coronavirus-contains-hiv-insertions-stoking-fears-over-artificially created-bioweapon Not coordinated URL: https://naturalnews.com/2020-02-03-the-coronavirus-was-engineered-by-scientists-in-a-lab.html

According to the statistics provided by Information Tracer, one URL, which we labeled “Definitely Coordinated”, falls squarely in the “definitely coordinated campaign” category because it exceeds average values on all metrics. A second URL, which we labeled “Not Coordinated”, falls in the “definitely not a coordinated campaign” range because all of its statistics are less than or equal to average values. A third URL, which we labeled “Possibly Coordinated”, has one measure that substantially exceeded average values, thus indicating that it *could* be part of a campaign, while two other statistics did not exceed average values. Its statistics lie on both sides of the suggested cutoffs used to separate campaigns from non-campaigns and are intended to indicate that it *might* be part of a campaign—i.e., the gist is that it is *possibly* part of a campaign. All URLs were labeled as misinformation by Information Tracer; the question for participants was whether they were part of a *coordinated* misinformation campaign.

### Procedure

At the beginning of the survey, participants were asked an attention check question (see Appendix E). Then, in a random order, participants either completed the individual differences measures, which were presented in a random order, or interacted with the system.

All subjects were randomly assigned to see either only the verbatim tutorial (the “verbatim” condition) or both the verbatim and gist tutorials (the “verbatim + gist” condition). Subjects were also randomly assigned to assess one of the three URLs described in the materials section. Participants used the Information Tracer system to track the spread of the assigned URL over multiple social media platforms and to decide whether it was part of a possible coordinated misinformation campaign based on the metrics described in the Materials section. Upon completing the tutorial, participants answered both gist judgment items and were asked to report verbatim metrics, described in the Instruments section. Participants were then asked to use Information Tracer to explore the system’s description of, and statistics about, the linked webpage. They then completed another set of gist judgments and reported verbatim metrics, this time for the assigned URL. After completing the gist and verbatim items, participants were asked to complete the system utility evaluation questionnaire. Evaluation questionnaire items were presented in a random order, then open–ended feedback questions from the system’s designers were presented. Participants then completed the demographics portion of the survey (see Appendix F) and received their MTurk completion code to receive payment.

### Analysis

#### System utility evaluation questionnaire

The 18 Likert-type items we developed to assess participants’ perceptions of discovery, explainability, and interpretability were scored on a 7-point scale ranging from − 3 (strongly disagree)to + 3 (strongly agree). Reverse-coded items were reverse-scored during analysis to maintain consistency in the direction of the response (e.g., a − 2 for a reverse-coded item would reflect the same level of disagreement as a +2 for a standard item). In addition, an attention check item was included in this questionnaire (see Appendix E).

#### Individual differences questionnaire

All standardized measures, including the Cognitive Reflection Test scales (combined into a single score), the Subjective Numeracy Scale, and the objective numeracy test, were scored according to the guidelines and scoring protocols established by their respective source materials. For the custom items related to use of formal methods, machine learning, and artificial intelligence in work, we calculated the average score across these items to create a composite measure. Finally, self-reported data science expertise was captured using a single question with multiple answer choices. Here, we utilized the highest level of expertise endorsed by the participant as their score.

#### Individual difference-system utility relationships

We first conducted Pearson correlations to examine bivariate relationships between all variables in our dataset. We next conducted linear regressions to examine whether predictors of work experience (the self-reported work experience scale), numerical ability (Lipkus et al., 2001), subjective perception of numerical ability (Fagerlin et al., 2007), and cognitive reflection (Frederick, 2005; Thomson and Oppenheimer, 2016) each accounted for unique variance in predicting system utility.

Finally, we conducted a two-way analysis of variance (ANOVA) in order to test the hypothesis that a gist intervention would have a positive impact on user judgments of campaign coordination. Our design was 2 (gist condition: verbatim vs gist + verbatim) x 3 (URL: Not Coordinated, Possibly Coordinated, or Coordinated), such that the gist condition and the URL shown were used as independent variables in the analysis, with coordinated campaign likelihood ratings of URLs as the dependent variable.

## Results

### Bivariate correlations

**Table 4:** Descriptive statistics and Pearson correlations for study variables

Variable	M (SD)	1	2	3	4	5	6	7	8
1. log(Reported breakout score)	1.41(1.06)	–							
2. log(Reported average tweets)	1.25(1.31)	$$0.65^{***}$$	–						
3.Reported % tweets	18.18(14.03)	$$0.39^{***}$$	$$0.43^{***}$$	–					
4. % Verbatim questions correct	0.76(0.41)	$$-0.61^{***}$$	$$-0.67^{***}$$	$$-0.33^{***}$$	–				
5. URL coordination rating$${^\text{a}}$$	$$-0.18 (1.41)$$	$$0.21^{**}$$	0.09	$$0.18^{**}$$	$$-0.14^{*}$$	–			
6. Interpretability$$^{\text{b}}$$	0.96(1.71)	$$-0.13$$	$$-0.08$$	$$-0.01$$	$$0.20^{**}$$	$$-0.08$$	–		
7. Explainability$$^{\text{b}}$$	0.63(1.74)	$$-0.08$$	$$-0.03$$	0.01	$$0.15^{*}$$	$$-0.09$$	$$0.74^{***}$$	–	
8. Discovery$${^\text{b}}$$	1.29(1.64)	$$-0.18^{*}$$	$$-0.19^{**}$$	$$-0.06$$	$$0.31^{***}$$	$$-0.02$$	$$0.73^{***}$$	$$0.57^{***}$$	–
9. Factual accuracy$${^{b}}$$	0.49(1.92)	0.01	0.04	0.05	0.06	$$-0.13$$	$$0.57^{***}$$	$$0.58^{***}$$	$$0.49^{***}$$
10. CRT$${^\text{c}}$$	0.64(0.32)	$$-0.42^{***}$$	$$-0.44^{***}$$	$$-0.32^{***}$$	$$0.55^{***}$$	$$-0.15^{*}$$	$$0.18^{**}$$	$$0.17^{*}$$	$$0.26^{***}$$
11. CRT1$${^\text{c}}$$	0.65(0.40)	$$-0.34^{***}$$	$$-0.38^{***}$$	$$-0.21^{**}$$	$$0.45^{***}$$	$$-0.08$$	$$0.16^{*}$$	$$0.16^{*}$$	$$0.22^{**}$$
12. CRT2$${^\text{c}}$$	0.64(0.31)	$$-0.43^{***}$$	$$-0.43^{***}$$	$$-0.36^{***}$$	$$0.55^{***}$$	$$-0.19^{**}$$	$$0.17^{*}$$	$$0.16^{*}$$	$$0.25^{***}$$
13. Objective numeracy$${^\text{c}}$$	0.79(0.28)	$$-0.58^{***}$$	$$-0.61^{***}$$	$$-0.36^{***}$$	$$0.69^{***}$$	$$-0.11$$	$$0.17^{*}$$	0.11	$$0.28^{***}$$
14.Subjective numeracy$${^\text{d}}$$	4.79(0.80)	0.11	0.09	$$0.15^{*}$$	$$-0.05$$	0.04	$$0.24^{***}$$	$$0.19^{**}$$	$$0.25^{***}$$
15. Subjective ability$${^\text{d}}$$	4.56(1.01)	$$0.15^{*}$$	$$0.18^{*}$$	$$0.21^{**}$$	$$-0.13$$	0.06	$$0.25^{***}$$	$$0.17^{*}$$	$$0.22^{**}$$
16. Subjective preference$${^\text{d}}$$	4.95(0.87)	0.05	$$-0.02$$	0.07	0.03	0.02	$$0.16^{*}$$	$$0.15^{*}$$	$$0.21^{**}$$
17.Self-reported work experience$${^{e}}$$	3.84(1.93)	$$0.42^{***}$$	$$0.48^{***}$$	$$0.23^{**}$$	$$-0.56^{***}$$	$$0.14^{*}$$	0.01	$$-0.02$$	$$-0.11$$

		9	10	11	12	13	14	15	16
10. CRT		$-0.07$	–						
11. CRT1		$$-0.10$$	$$0.91^{***}$$	–					
12. CRT2		$$-0.02$$	$$0.92^{***}$$	$$0.66^{***}$$	–				
13. Objective numeracy		$$-0.07$$	$$0.72^{***}$$	$$0.65^{***}$$	$$0.67^{***}$$	–			
14. Subjective numeracy		0.07	$$0.19^{**}$$	$$0.20^{**}$$	$$0.14^{*}$$	0.13	–		
15. Subjective ability		0.09	0.10	$$0.14^{*}$$	0.04	0.02	$$0.84^{***}$$	–	
16. Subjective preference		0.04	$$0.22^{***}$$	$$0.21^{**}$$	$$0.19^{**}$$	$$0.19^{**}$$	$$0.88^{***}$$	$$0.49^{***}$$	–
17. Self-reported work experience		$$0.15^{*}$$	$$-0.49^{***}$$	$$-0.44^{***}$$	$$-0.45^{***}$$	$$-0.56^{***}$$	0.12	$$0.30^{***}$$	$$-0.07$$

$${^{***}}$$ = $$p \, {\le} \, 0.001^{**}$$ = $$p \, {\le} \, 0.01$$. $$^{*}$$ = $$p \, {\le} \, 0.05$$. M, mean; SD, standard deviation; CRT, combined cognitive reflection test (Pennycook and Rand, 2019b); CRT1, 1st cognitive reflection test (Frederick, 2005); CRT2, 2nd cognitive reflection test (Thomson and Oppenheimer, 2016)

${^\text{a}}$ This measure used a 7-point Likert scale ranging from − 3 (strongly disagree) to + 3 (strongly agree)

${^\text{b}}$ These measures used a 9-point Likert scale ranging from − 4 (strongly disagree) to +4 (strongly agree)

${^\text{c}}$ These scales were scored by the proportion of items that subjects answered correctly

${^\text{d}}$ These measures used a 6-point Likert scale ranging from 1 (e.g., “not at all”) to 6 (e.g., “extremely”). Scale endpoint labels varied somewhat between items (see Fagerlin et al. (2007) for details)

${^\text{e}}$ This measure used a 7-point Likert scale ranging from 0 (subject indicated no prior experience with artificial intelligence, data science of machine learning) to 6 (subjected indicated extensive prior experience)

Pearson correlation results are shown in Table 4.

#### Verbatim and gist measures

*Verbatim measures* We found significant positive correlations between all verbatim metrics reported in Information Tracer. When subjects answered these questions incorrectly, they tended to overreport the risks of campaign coordination, as indicated by a significant negative correlation between these metrics and the proportion of questions answered correctly. They were also more likely to overreport the risks if they reported more data science work experience. Furthermore, subjects were more likely to answer these questions correctly if they were more numerate and if they scored more highly on the cognitive reflection test. Finally, subjects who answered these questions correctly reported a greater ability to use the system to gain new insights.

*Gist ratings* Subjects were more likely to report that URLs were part of a coordinated campaign if they reported a higher breakout score and if more tweets were generated by the top 10% of accounts. Additionally, when subjects answered verbatim questions incorrectly, they were slightly, but significantly, more likely to categorize URLs as part of a campaign. Unlike the verbatim measures, we did not detect significant associations between numeracy measures and URL ratings. Cognitive reflection was only slightly associated with URL coordination ratings, and only for the items introduced by Thomson and Oppenheimer (2016). As with the verbatim items, subjects reporting more data science work experience were slightly more likely to consider URLs as part of a coordinated campaign.

#### System utility measures

All system utility measures were significantly intercorrelated, indicating related, yet distinct, constructs.

*Interpretability, explainability, and discovery* Subjects who did not answer verbatim questions correctly also found the system less interpretable, less explainable, and less useful for generating new insights. On the other hand, subjects who were more reflective and more numerate, and who reported more prior data science experience found the system to be more useful across virtually all metrics (although the effect of objective numeracy on explainability was not statistically significant).

*Factual accuracy judgments* Unlike the other system utility measures, only self-reported work experience was significantly associated with endorsements of the system as helping to assess factual accuracy of URLs.

#### Cognitive reflection and numeracy

Measures of cognitive reflection, objective numeracy and subjective numeracy were significantly intercorrelated, with especially strong correlations between objective numeracy and cognitive reflection (although see Liberali et al. (2012)). We observed weaker correlations between subjective preference for numbers and objective numeracy measures. In contrast, subjective mathematical ability ratings appear to be largely uncorrelated with objective numeracy.

#### Self-reported work experience

Subjects who reported more experience with data science tended to have lower objective numeracy and cognitive reflection scores, but higher subjective assessments of their own mathematical abilities.

### Predictors of system utility

We next examined which factors predicted ratings of system utility. To do so, we conducted linear regressions with interpretability, explainability, discovery, and factual accuracy facilitation judgments as the dependent variables. Our aim was to determine if predictors such as work experience, numerical ability, and subjective perception of numerical ability (controlling for actual ability) accounted for unique variance despite being correlated with one another in predicting these dependent variables. We did not include cognitive reflection in these analyses due to the strong multicollinearity between the CRT and the Lipkus objective numeracy scale that we observed. (We conducted an exploratory factor analysis with maximum likelihood factor extraction and found that all objective numeracy and CRT items loaded on a single factor.) Results of these regression analyses are shown in Table 5. Participants’ judgments of interpretability and discovery increased with both objective numeracy and subjective mathematical ability. We did not observe significant associations between these measures of individual differences and explainability ratings or assessments of whether the system facilitated factual accuracy judgments.

**Table 5:** Multiple regressions predicting interpretability, explainability, discovery, and factual accuracy ratings

	*B*	SE	$\beta$	*t*	*p*	95% CI	
						Lower	Upper
	Interpretability						
Gist condition (Gist + Verbatim)	0.37	0.23		1.59	0.11	$-0.09$	0.83
Self-reported work experience${^\text{a}}$	0.03	0.08	0.04	0.42	0.68	$-0.12$	0.19
Objective numeracy${^\text{b}}$	1.03	0.51	0.17	2.00	$0.047^{*}$	0.02	2.04
Subjective ability$${^\text{c}}$$	0.38	0.14	0.23	2.65	$$0.009^{**}$$	0.10	0.66
Subjective preference${^\text{c}}$	0.07	0.16	0.03	0.42	0.68	$-0.25$	0.38
(Intercept)	$-2.22$	0.85		$-2.60^{*}$	0.01	$-3.91$	$-0.54$
	Explainability						
Gist condition (Gist + Verbatim)	0.15	0.24		0.63	0.53	$-0.33$	0.63
Self-reported work experience${^{a}}$	$-0.01$	0.08	$-0.01$	$-0.09$	0.93	$-0.17$	0.16
Objective numeracy${^\text{b}}$	0.55	0.54	0.09	1.01	0.31	$-0.52$	1.61
Subjective ability${^\text{c}}$	0.24	0.15	0.14	1.56	0.12	$-0.06$	0.53
Subjective preference${^\text{c}}$	0.15	0.17	0.07	0.89	0.38	$-0.18$	0.48
(Intercept)	$-1.66$	0.90		$-1.85$	0.07	$-3.43$	0.11
	Discovery						
Gist condition (Gist + Verbatim)	0.30	0.22		1.35	0.18	$-0.14$	0.73
Self-reported expertise${^\text{a}}$	$-0.03$	0.07	$-0.03$	$-0.34$	0.74	$-0.17$	0.12
Objective numeracy$${^\text{b}}$$	1.40	0.49	0.24	$$2.87^{**}$$	0.005	0.44	2.35
Subjective ability${^\text{c}}$	0.31	0.14	0.19	$2.29^{*}$	0.02	0.04	0.58
Subjective preference${^\text{c}}$	0.15	0.15	0.08	0.97	0.33	$-0.15$	0.44
(Intercept)	$-2.00$	0.81		$-2.48^{*}$	0.01	$-3.60$	$-0.41$
	Factual accuracy						
Gist (Gist + Verbatim)	0.37	0.27		1.38	0.17	$-0.16$	0.91
Self-reported expertise${^\text{a}}$	0.15	0.09	0.16	1.69	0.09	$-0.03$	0.34
Objective numeracy${^\text{b}}$	0.01	0.60	0.00	0.02	0.99	$-1.18$	1.19
Subjective ability${^\text{c}}$	0.03	0.17	0.02	0.18	0.86	$-0.30$	0.36
Subjective preference${^\text{c}}$	0.12	0.19	0.05	0.64	0.52	$-0.25$	0.49
(Intercept)	$-1.02$	1.00		$-1.02$	0.31	$-2.99$	0.95

B, regression coefficient; SE, standard error; $\beta$, standardized regression coefficient; 95% CI, 95% confidence interval. The reference class for these regressions was the verbatim tutorial condition with the “Not Campaign” URL

$^{a}$ This measure used a 7-point Likert scale ranging from 0 (subject indicated no prior experience with artificial intelligence, data science of machine learning) to 6 (subjected indicated extensive prior experience)

$^{b}$ This scale were scored by the proportion of items that subjects answered correctly

$^{c}$ These measures used a 6-point Likert scale ranging from 1 (e.g., “not at all”) to 6 (e.g., “extremely”). Scale endpoint labels varied somewhat between items (see Fagerlin et al. (2007) for details)

*=*p* < 0.05, **=*p* < 0.01

### Impact of gist condition on ability to discriminate URLs

We next performed a two-way ANOVA using ratings of URLs as part of misinformation campaigns as the dependent variable and the gist intervention, and the URL shown as independent variables. We found a significant main effect of the URL shown, $F(2,199) = 31.05, p <.001, \eta ^2_p = 0.24$, and a significant interaction between the URL shown and the gist condition, $F(2,199) = 3.74, p = 0.03, \eta ^2_p = 0.04$. We did not detect a significant main effect of gist $F(1,199) = 0.35, p = 0.55, \eta ^2_p = 0.00$. Balance tests showed that subjects’ cognitive reflection, objective and subjective numeracy, and self-reported expertise did not vary significantly between conditions. Mean differences between conditions are shown in Table 6. Results show that differences between the “Not Coordinated” URL and the other two URLs were larger when subjects were shown the gist tutorial compared to when they were not. Thus, the gist condition appears to have increased subjects’ abilities to discriminate between URLs that might have been coordinated from one that was not.

**Fig. 2:**
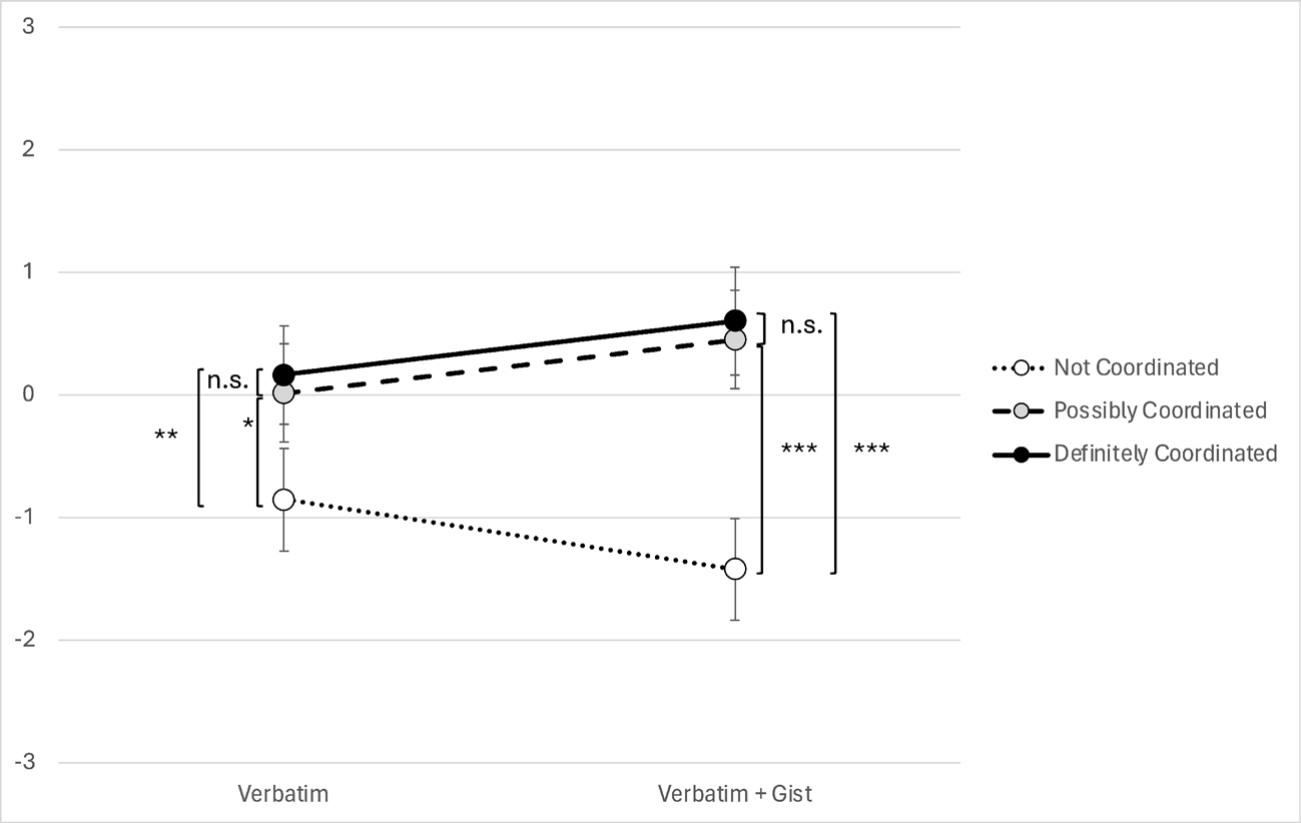
Participant agreement that a given URL is part of a coordinated misinformation campaign. Ratings range from strongly disagree (−3) to strongly agree (+ 3). Error bars are 95% confidence intervals. ***= *p*$\le$ 0.001, **= *p*$\le$ 0.01, *= *p*$\le$ 0.05, n.s., not significant. *P*-values are calculated after applying a Tukey HSD test for multiple comparisons

The gist intervention led to participants more “correctly” rating the URL with clear indicators of being part of a coordinated misinformation campaign as being part of a campaign and rating the URL with clear indicators it was not part of a campaign as not being part of a campaign. However, as seen in Fig. 2, when assessing the most ambiguous URL (URL Two), which did not have clear indicators in either direction, those presented with the gist intervention were more likely than the other participants to decide that the URL was part of this kind of campaign.

**Table 6:** Tukey HSD test results comparing URL coordination rating pairs

First condition		Second condition			95% CI				
coordinated?	Gist?	coordinated?	Gist?	*M*	Lower	Upper	SE	*t*	$p_\text {tukey}$
Not	No	Not	Yes	0.57	− 0.30	1.43	0.30	1.88	0.42
		Possibly	No	− 0.87	− 1.73	− 0.02	0.30	− 2.93	0.04*
			Yes	− 1.30	− 2.16	− 0.45	0.30	− 4.39	< 0.001***
		Definitely	No	− 1.02	− 1.87	− 0.16	0.30	− 3.42	0.01**
			Yes	− 1.46	− 2.35	− 0.56	0.31	− 4.68	< 0.001***
	Yes	Possibly	No	1.44	0.59	2.29	0.30	4.88	< 0.001***
			Yes	− 1.87	− 2.72	− 1.02	0.30	− 6.35	< 0.001***
		Definitely	No	1.58	0.73	2.43	0.30	5.37	< 0.001***
			Yes	− 2.02	− 2.91	− 1.13	0.31	− 6.55	< 0.001***
Possibly	No	Possibly	Yes	− 0.43	− 1.27	0.40	0.29	− 1.49	0.67
		Definitely	No	0.15	− 0.69	0.98	0.29	0.50	1.00
			Yes	− 0.59	− 1.46	0.29	0.31	− 1.92	0.39
	Yes	Definitely	No	− 0.29	− 1.12	0.55	0.29	− 0.99	0.92
			Yes	0.15	− 0.73	1.03	0.31	0.50	1.00
Definitely	No	Definitely	Yes	− 0.44	− 1.32	0.44	0.31	− 1.45	0.70

***= *p* ≤ 0.001. **= *p* ≤ 0.01. *= *p* ≤ 0.05. M, mean difference; SE, standard error. $p_\text {tukey}$ = *p*-value after adjusting for post-hoc comparisons using the Tukey HSD test

In order to rule out the possibility that subjects’ responses to URL coordination ratings might have been driven by misperceptions of Information Tracer metrics, we next conducted an analysis in order to verify that our results replicated when controlling whether these items were answered correctly. Results are shown in Table 7.

**Table 7:** ANCOVA Predicting the effect of the gist tutorial and URL shown on campaign coordination ratings, controlling for whether verbatim item responses were correct

	SS	df	Mean Square	F	p	$\eta ^2_p$
Breakout Score Correct?	5.53	1	5.53	3.82	0.05	0.02
Average Tweets per User Correct?	3.67	1	3.67	2.53	0.11	0.01
Percent Tweets from top 10% of Users Correct?	1.82	1	1.82	1.26	0.26	0.01
URL	100.50	2	50.25	34.66	<.001***	0.26
Gist	1.28	1	1.28	0.88	0.35	0.00
URL * Gist	12.46	2	6.23	4.30	0.02*	0.04
Residuals	284.16	196	1.45			

Note. *** =* p* < 0.001. ** =* p* < 0.01. * =* p* < 0.05. SS = Sum of Squares. df = degrees of freedom

Verbatim measures were coded as dummy variables with 1 = correct and 0 = incorrect

We also detected a significant interaction between gist and URL shared when including measures of cognitive reflection, numeracy, and self-reported work experience as covariates, $F(2,192) = 4.08, p = 0.02, \eta ^2_p = 0.04$, (none of these individual difference measures were statistically significant upon their inclusion).

#### Distinct contributions of self-reported interpretability

Although we did not observe a direct effect of individual differences on URL coordination ratings, we did observe an effect of numeracy on interpretability ratings. We therefore performed a *post hoc* analysis to determine whether subjects who endorsed Information Tracer as interpretable were better able to use the tool to discriminate between URLs. To do so, we conducted another multiple regression under the hypothesis that we would find a significant interaction between URL type and interpretability ratings beyond the effects of the URL and gist condition that we observed in the previous ANOVA. Results, shown in Table 8, demonstrate that subjects who found Information Tracer output to be interpretable were better able to discriminate between URLs, beyond the effects of the gist intervention. Specifically, subjects reporting increased interpretability rated the “Not Coordinated” URL lower and rated the other two URLs higher on the coordinated campaign scale.

**Table 8:** Regression model including interpretability and its interaction with URL shown

Model		*B*	SE	*t*	*p*
H$$_{0}$$	URL (Definitely campaign)	1.02	0.30	3.42	< 0.001$${^{***}}$$
	URL (Possibly campaign)	0.87	0.30	2.92	$$0.004^{**}$$
	(Gist + Verbatim)	$-0.57$	0.30	$-1.88$	0.06
	URL (Definitely campaign) * (Gist + Verbatim)	1.01	0.43	2.35	$0.02^{*}$
	URL (Possibly campaign) * (Gist + Verbatim)	1.00	0.42	2.39	$0.02^{*}$
	(Intercept)	$$-0.86$$	0.22	$$-3.99$$	< $$0.001^{***}$$
H$$_{1}$$	Interpretability$${^{a}}$$	$$-0.27$$	0.08	$$-3.51$$	< 0.001$$^{***}$$
	URL (Definitely campaign)	0.72	0.31	2.33	$0.02^{*}$
	URL (Possibly campaign)	0.68	0.30	2.25	$0.03^{*}$
	(Gist + Verbatim)	$-0.47$	0.30	$-1.59$	0.11
	Interpretability * URL (Definitely campaign)	0.38	0.12	3.07	$$0.002^{**}$$
	Interpretability * URL (Possibly campaign)	0.28	0.12	2.35	$0.02^{*}$
	URL (Definitely campaign) * (Gist + Verbatim)	0.88	0.42	2.10	$0.03^{*}$
	URL (Possibly campaign) * (Gist + Verbatim)	0.90	0.41	2.18	$0.03^{*}$
	(Intercept)	$$-0.67$$	0.22	$$-3.10$$	$$0.002^{**}$$

$$^{***}$$= *p* ≤ 0.001. $$^{**}$$= *p* ≤ 0.01. $$^{*}$$= *p* ≤ 0.05. *B*, regression coefficient; SE, standard error. H$$_{0}$$ = Baseline model containing the same terms as the ANOVA. H$$_{1}$$ = Model adding effects of self-reported interpretability and its interaction with the URL shown. The model reference class is the “Not coordinated” URL in the Verbatim condition

$^{a}$ This measure used a 9-point Likert scale ranging from − 4 (Strongly disagree) to + 4 (Strongly agree)

## Discussion

The primary goal of Information Tracer is to help users to identify URLs that are artificially amplified as part of a coordinated misinformation campaign. To do so, the tool provides users with three metrics that are intended to be indicative of such coordination. Results showed that at least two of these metrics—breakout score and the proportion of tweets generated by the top 10% most active accounts—do indeed appear to help subjects identify such campaigns. However, the effects of adding gist explanations to the verbatim metrics, shown in Fig. 2, further indicated that the metrics were not very helpful without an explanation of their gist.

### Gist intervention enables better discrimination between URLs

Beyond the small effects of these verbatim metrics, we found that providing subjects with a brief gist tutorial interpreting these metrics helped them to better discriminate URLs that might have been indicative of a coordinated campaign from those that were not. As predicted, we observed a significant interaction between the gist intervention and the specific URL presented. When the URL was clearly part of a coordinated campaign, the gist intervention increased subjects’ rating of it as such. Similarly, when the URL was clearly NOT part of a coordinated campaign, the gist intervention reduced this rating. On the other hand, we did not observe significant differences between the “Definitely Coordinated” and “Possibly Coordinated” URLs. These findings are consistent with fuzzy-trace theory’s tenet that gists are encoded into imprecise categories that emphasize “some” versus “none” distinctions (in this case, whether or not a URL was possibly part of a coordinated campaign) (Reyna, 2012; Broniatowski and Reyna, 2018). Even when controlling for verbatim responses, we observed a significant interaction of gist and URL shared, meaning that our results cannot be explained by differences in verbatim assessments of system outputs.

The interaction between the gist intervention and the type of URL provided to participants with decision correctness suggests that tutorials that are designed to communicate the gist of automated tools may be beneficial. As fuzzy-trace theory suggests, providing a gist tutorial and empowering users to find the meaning of Information Tracer’s output improved participants’ discernment.

### Numeracy and mathematical self-confidence improve discernment via interpretability

Automated systems are unlikely to be adopted or widely used if subjects do not perceive them to be useful or interpretable. Our results indicate that subjects with higher objective numeracy and higher ratings of their own mathematical abilities—i.e., higher mathematical self-confidence—(Peters et al., 2019) were more likely to report being able to make sense of Information Tracer’s outputs and more likely to find it useful for discovering new insights. Furthermore, we found that these interpretability endorsements were significantly associated with improved URL discernment beyond the effects of the gist intervention (we found similar results when using the discovery scale instead of the interpretability scale, and indeed, these two quantities were strongly correlated). Thus, it appears that the gist tutorial, subjects’ objective numeracy skills, and subjects’ subjective assessments of their mathematical abilities all play distinct roles in improving users’ judgments. In contrast, self-reported prior work experience—which displayed a pattern consistent with potential overconfidence (i.e., high subjective numeracy but low objective numeracy)—did not appear to significantly predict performance.

### Limitations

Since our study was conducted using the experiences of online microworkers, future work might examine whether the relationships found in this work replicate in highly skilled, professional computer scientists and/or non-computer-scientist subject matter experts, such as journalists on the misinformation beat.

In this work, participants were presented with one or both of the two system tutorials. Due to limitations in our data collection, we were unable to track active reading time as opposed to time spent with the longer tutorial open in a background window or tab. We are therefore unable to account for any confound introduced by the difference in length and time participants spent reading between tutorials. Future studies might incorporate more effective time-tracking measures to make this comparison and analysis possible. However, this limitation is mitigated by the fact that subjects given the gist tutorial also encountered the verbatim tutorial, meaning that the relatively poor performance of subjects in the verbatim condition cannot be attributed to fatigue.

## Conclusions

Our findings align with fuzzy-trace theory, suggesting that gist-based interventions like the one employed here can improve decision making when users interact with automated systems.

Specifically, these results suggest that gist mental representations, interpretability, and meaning all play key roles in automated system user performance. We find that there are multiple paths to meaning that designers can take advantage of when attempting to improve the uptake and use of automated systems. On the one hand, users who were more numerate reported being better able to make sense of system output. Beyond mathematical skill, users who were more confident in their mathematical abilities also reported enhanced interpretability. This, in turn, translated to better discernment of coordinated from non-coordinated URLs. On the other hand, subjects who were given a brief tutorial explaining how output metrics should be interpreted also experienced enhanced discernment in a manner that was independent of their mathematical abilities or confidence. Thus, our findings suggest ways to tailor system output to users who differ in their confidence and abilities and suggest that numeracy, although helpful for making sense of system output, is not an absolute requirement. Rather, designers may incorporate tutorials or similar tools that utilize gist representations of system output alongside verbatim tutorials. This might include emphasizing the “bottom line” of each section of a tutorial by explicitly highlighting important metrics and how they relate to baseline values around which categorical distinctions turn, or by designing visualizations and other output that help users to interpret the information in its context—i.e., to communicate the gist of the information. In short, interventions that enhance meaning–making can improve performance.

## Figures and Tables

**Fig. 3:**
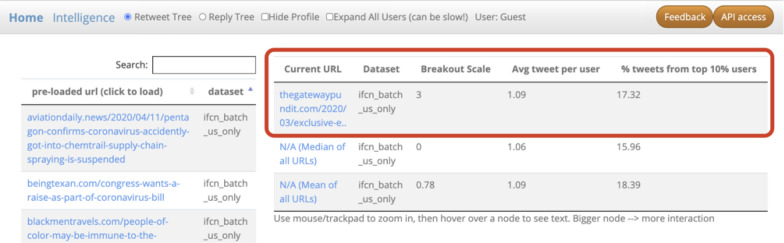
Image from verbatim tutorial highlighting URL information

**Fig. 4:**
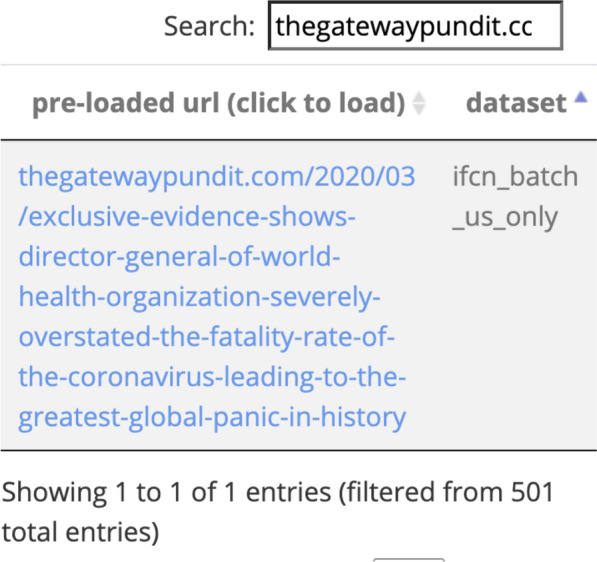
Image from verbatim tutorial demonstrating how to search for a URL

**Fig. 5:**
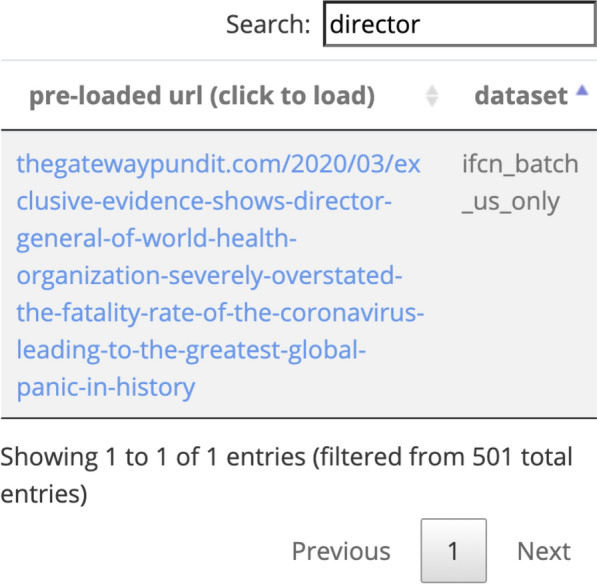
Image from verbatim tutorial demonstrating how to search a keyword

**Fig. 6:**
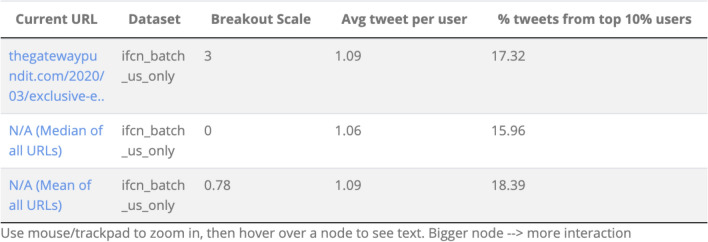
Image from verbatim tutorial zooming in on URL information

**Fig. 7:**
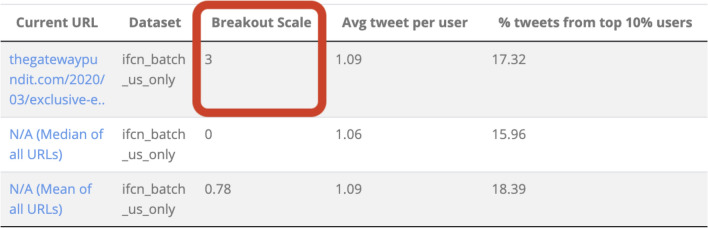
Image from verbatim tutorial highlighting breakout scale detail

**Fig. 8:**
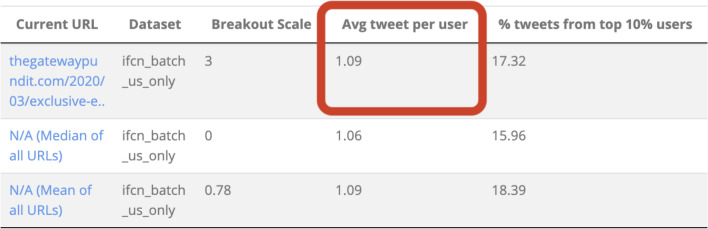
Image from verbatim tutorial highlighting average tweets per user detail

**Fig. 9:**
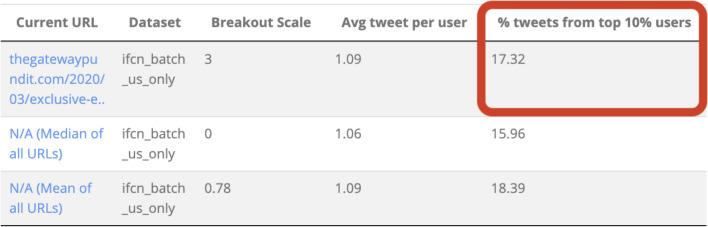
Image from verbatim tutorial highlighting tweet proportion detail

**Fig. 10:**
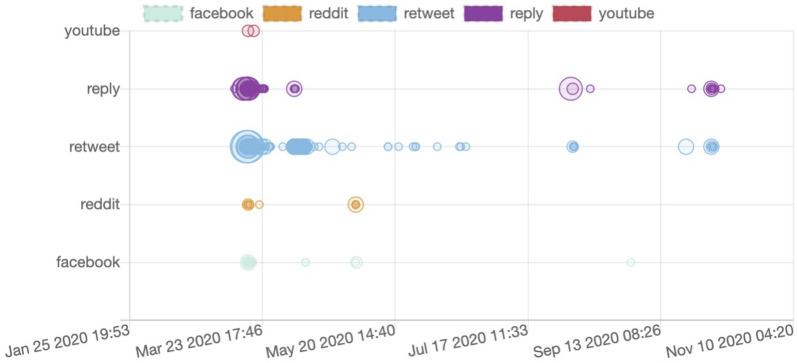
Plot image from verbatim tutorial showing engagement on various social media with URL over time

**Fig. 11:**
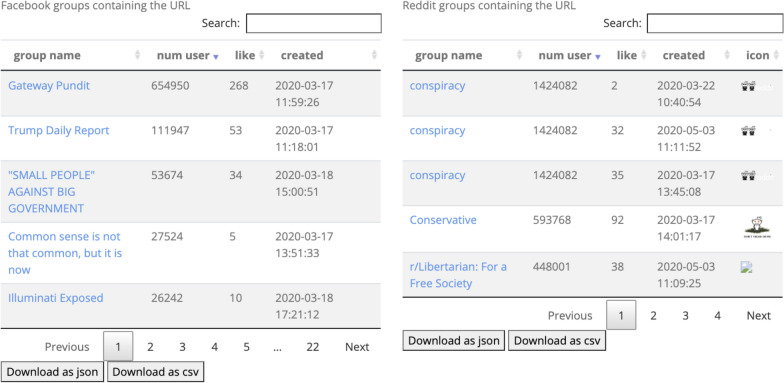
Tables from verbatim tutorial showing engagement with URL on Facebook and Reddit over time

**Fig. 12:**
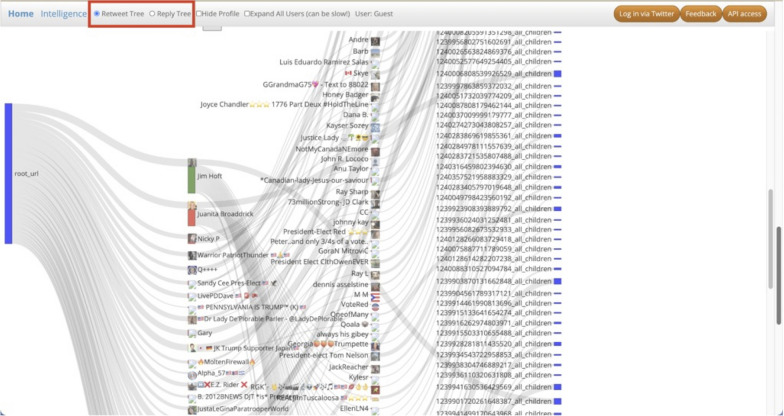
Screenshot of verbatim tutorial showing retweet tree

**Fig. 13:**
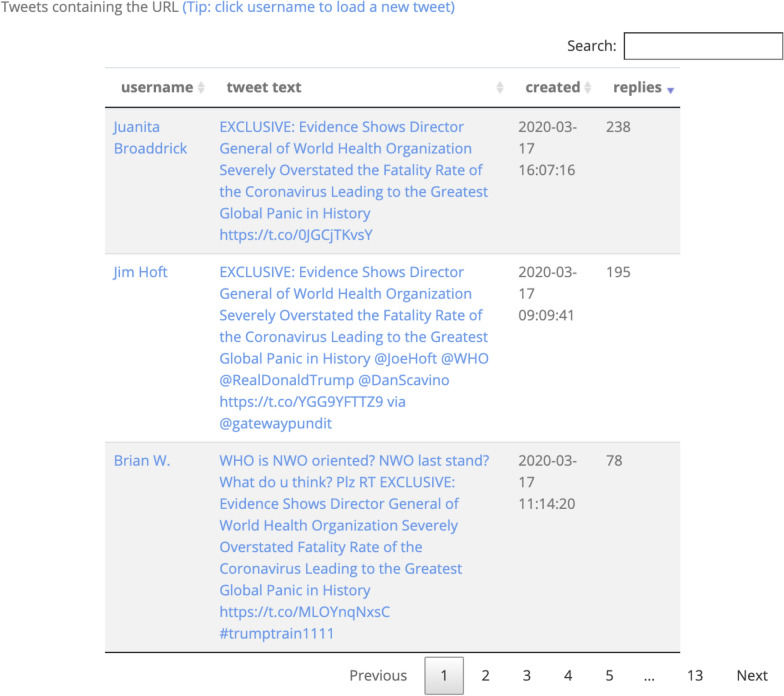
Table from verbatim tutorial highlighting sample tweets containing URL

**Fig. 14:**
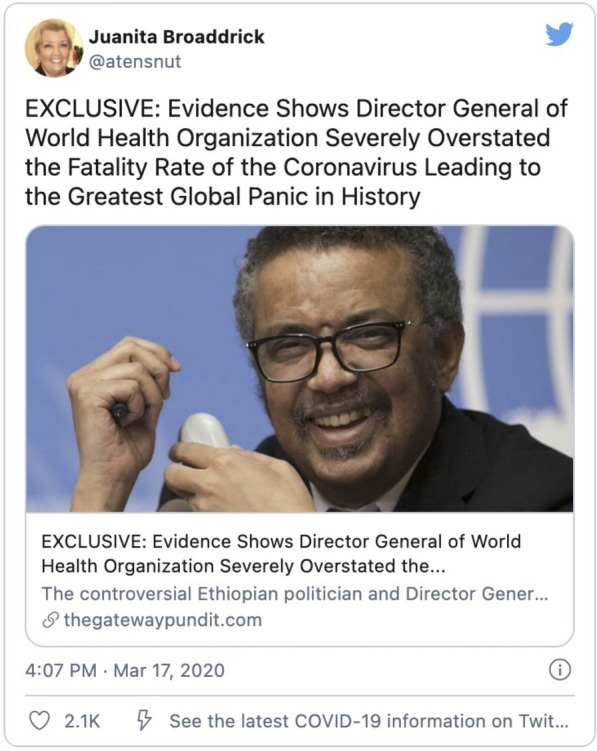
Screenshot from verbatim tutorial of tweet containing URL

**Fig. 15:**
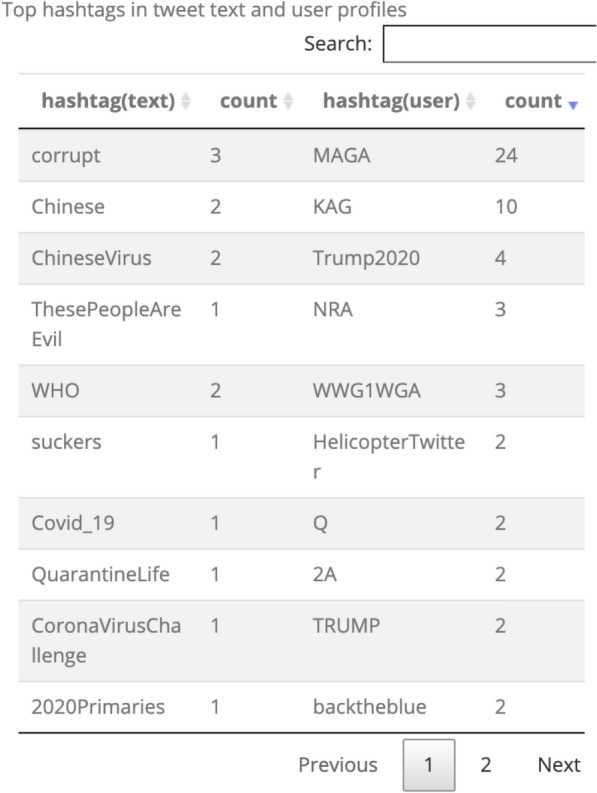
Screenshot from verbatim tutorial of tweet containing URL

**Fig. 16:**
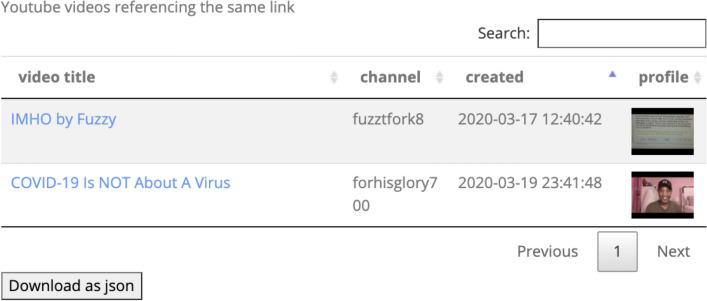
Table from verbatim tutorial highlighting YouTube videos mentioning URL

**Fig. 17:**
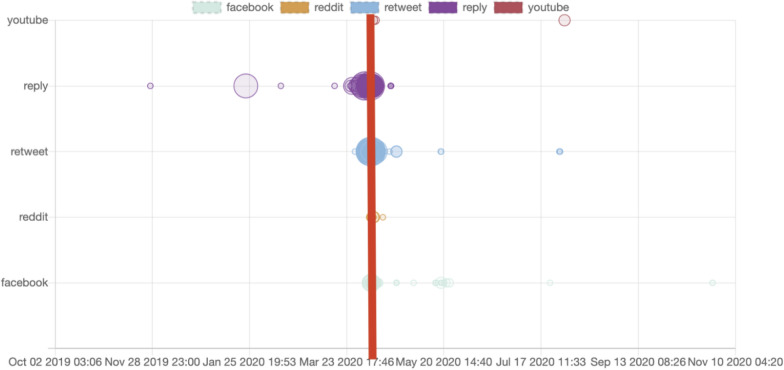
Image from gist tutorial showing potential post coordination

**Fig. 18:**
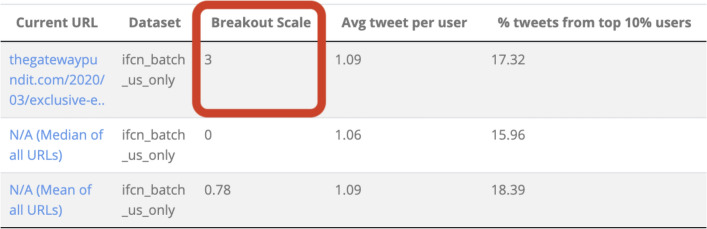
Image from gist tutorial showing breakout scale detail

**Fig. 19:**
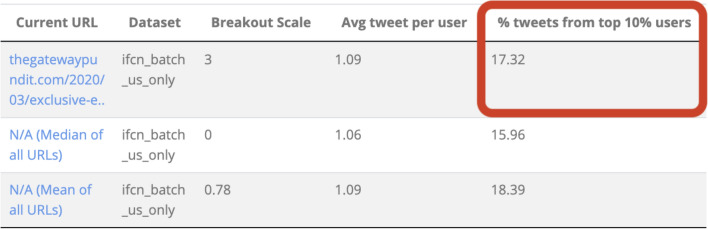
Image from gist tutorial showing tweet proportion detail

**Fig. 20:**
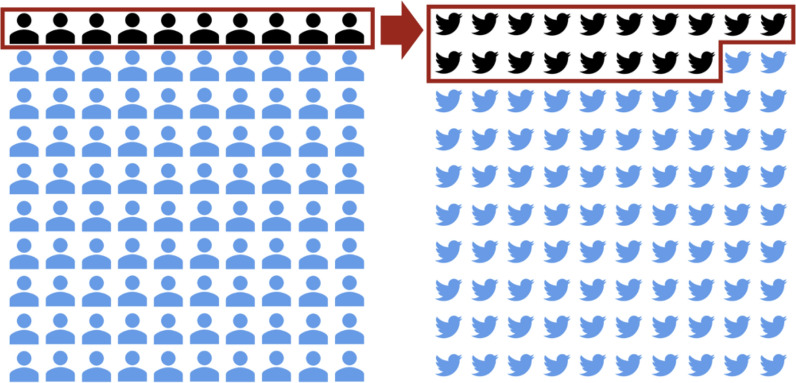
Image from gist tutorial showing average Tweet proportion

**Fig. 21:**
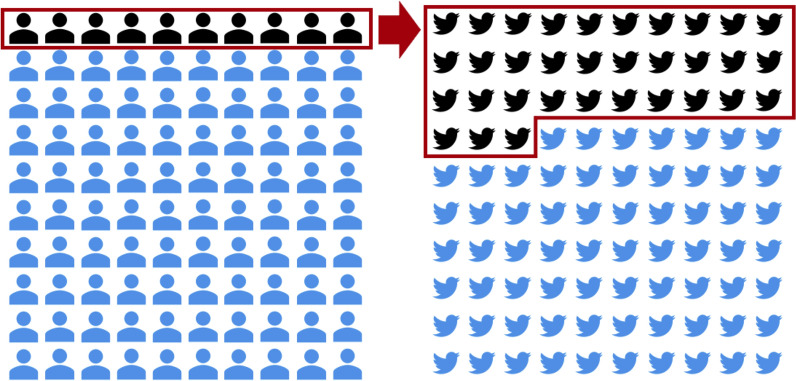
Image from gist tutorial showing higher-than-average tweet proportion

**Fig. 22:**
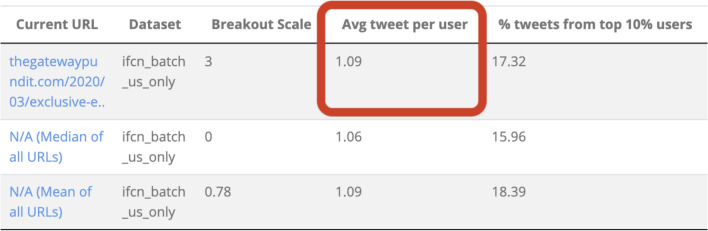
Image from gist tutorial showing “[Average] Tweet per User” detail

## Data Availability

The datasets used and analyzed during the current studies are available from the corresponding author on reasonable request.

## Appendix A: Full list of outputs elicited from information tracer

- What is the headline of the article you were looking at?
- How many original posts were there on Facebook about this article?
- How many replies did the most-replied-to tweet about this URL get?
- What was the breakout scale score?
- What was the average number of tweets per user about this URL?
- What percent of tweets about this URL came from the top 10% of users?
- What is the top hashtag associated with the user “[see below]” (@[see below])? *If there are multiple top hashtags in a tie, please list them all.*
- How many YouTube videos reference this URL?
- What are the dates of the earliest and most recent posts about this URL? *Please use the format mm/dd/yyyy.*
  - Earliest
  - Latest

## Appendix B: System utility evaluation free response

We included free-response items focused primarily on the functionality of the system and how users felt the system could be improved and were not included in our analysis. They were as follows:

- *What aspects/features do you like most about the system?*
- *What aspects/features do you think the system needs to improve on?*
- *Is there anything that you wish the system was able to do? Think of this like a wishlist of features.*
- *Do you have any feedback to improve the user interface of the system? Were there any technical issues that prevented you from completing your task? (For example, the system was not available/was too slow/showed errors)*

## Appendix C: Full verbatim tutorial

Here is a video about how information tracer works. Watching the video is not required, but you may find it helpful. A short text tutorial also follows. Feel free to use either the video, the text tutorial or both.

[embedded video: https://www.youtube.com/watch?v=iNlWUlE7dCY]

Although watching the video is not required, we’d like to know if you did.

Please indicate how much of the video you watched in the format (minutes:seconds). (If you did not watch the video, please enter “0:00”.):

If you did not watch the video, please check the box below.

( ) I did not watch the video

### Information tracer tutorial

To make sure you’re comfortable with the Information Tracer system, you may follow this short tutorial. The information given here is also shown in a video tutorial at start of this page. In this tutorial, you’ll be looking at the web link (or URL):

URL: http://thegatewaypundit.com/2020/03/exclusive-evidence-shows-director-general-of-world-health-organization-severely-overstated-the-fatality-rate-of-the-coronavirus-leading-to-the-greatest-global-panic-in-history

As a reminder, all of the web links (URLs) that you will look at during this study are “fake news,” or misinformation.

First, please open this link in another window:

URL: https://beta.informationtracer.com/

### Choosing an article URL

The Gateway Pundit article should be the default URL shown in the table on the right (Fig. 3) when you open Information Tracer.

If it is not, copy and paste this URL in the “Search” text box (Fig. 4): URL: thegatewaypundit.com/2020/03/exclusive-evidence-shows-director-general-of-world-health-organization-severely-overstated-the-fatality-rate-of-the-coronavirus-leading-to-the-greatest-global-panic-in-history

Later, you can use this text box to search for keywords and find relevant URLs—in the picture below (Fig. 5), we have searched for “director” and found this same article. In this tutorial, you will only be using the Gateway Pundit article.

### Social media statistics

To the right, you can see some information, highlighted with a red box below, about how often the URL was shared across social media (Fig. 3).

In the next image (Fig. 6), we will zoom in on the table in the red box.

Information Tracer provides three indicators of how much a URL was shared (Fig. 7):

- *Breakout scale* The number of other social media platforms with links pointing to the original post containing this URL.
  - A URL is said to “break out” on a platform if there are more than 100 interactions with the URL.
  - For example, if an article was only shared on Twitter and has fewer than 100 retweets and/or replies, the breakout scale is 0.
  - If an article is widely shared on Twitter (100+ retweets and/or replies), and there are fewer than 100 posts linking to any of these tweets on other platforms, the breakout scale is 1.
  - If the article was widely shared on Twitter and Facebook, and was posted once on Reddit but had zero comments, then the breakout scale is 2.
- *Average number of tweets per user* How many times an account in the conversation on Twitter shared this URL, on average (Fig. 8)
- *Percentage of tweets about this URL* that come from the top 10% of users with the most tweets in the conversation about this URL (Fig. 9)

You also see the median and mean (average) for these stats for the entire collection of URLs for comparison.

There is also a chart (Fig. 10) showing the timeline of users sharing and mentioning the URL on different platforms. The size of each bubble on the chart represents how big the discussion was in each post—the more comments, retweets, or likes, the larger the bubble is.

### Facebook and Reddit groups

Scroll down to the tables in the next row (Fig. 11). These tables tell you about the Facebook and Reddit groups in which this URL was posted most often. You can see the name and size of the group (or “subreddit”—a group on Reddit) the post was in, as well as how many likes the post got and when it was posted. Clicking a link under “group name” will take you to the post and let you see how the URL was mentioned in that group.

### Tweets

Near the bottom of the page is a visualization of who posted the URL on Twitter, and who retweeted or replied to those posts (Fig. 12). In the second column, you see original tweets about the URL; as you go right, you can see retweets of those, then retweets of those retweets, and so on until the end of each chain, where there are no more retweets.

On the top bar, you can change “Retweet Tree” to “Reply Tree” to see the same information for replies.

Hovering over an account picture lets you see the content of the tweets; clicking the picture will highlight that tweet’s path, making it easier to see in the chart (Fig. 13).

You can also see any of the original tweets by going to the table above (Fig. 14). It tells you the username and text of the tweet, when the tweet was posted, and how many replies it has. Clicking the username here will load the tweet a row up.

The table below lets you see the top hashtags associated with this tweet and the top hashtags associated with this user (Fig. 15).

### YouTube videos

In the bottom row, there’s a table of YouTube videos referencing the URL (Fig. 16). You can see the title of each video it was mentioned in, the channel it was posted to, and other relevant information.

## Appendix D: Full gist description

### The bottom line

The following features are signs of a potential coordinated misinformation campaign:

1. Social media accounts that post the same content at the same time display coordination (Fig. 17):
2. If the URL has a high breakout score, that means it got many comments, replies, or retweets on several social media platforms. A high score might indicate a coordinated misinformation campaign because the more platforms that an article is shared on, the higher the audience of users that can be targeted and manipulated. Most URLs have a breakout score of 1 or lower (Fig. 18).
3. When the % of tweets coming from the top 10% of users (the most active 10% of users in the conversation about this URL) is much higher than average, this could suggest a coordinated misinformation campaign because misinformation is more likely to come from a small number of active users (Fig. 19). Below (Fig. 20), we can see that the top 10% of users are contributing 18% of the tweets in the conversation. This is about average for this dataset. In the next image (Fig. 21), however, the amount of tweets that the top 10% of users made is much higher: 33%, or about a third. This higher proportion might indicate a misinformation campaign.
4. A higher-than-average number of tweets per user could mean that someone is attempting to artificially amplify the popularity of the URL, potentially indicating a coordinated misinformation campaign (Fig. 22).

## Appendix E: Attention Check Items

### Introduction

People are very busy these days and many do not have time to read the news. We are testing whether people read questions. To show that you’ve read this much, answer both “extremely interested” and “very interested.”

- Extremely interested
- Very interested
- Moderately interested
- Slightly interested
- Not interested at all

### System evaluation

I can answer this question correctly by clicking on Disagree.

## Appendix F: Demographics questionnaire

Now, we would like to ask you some questions about yourself.

1. What is your gender?
  - Male
  - Female
  - Other (FILL IN):
2. Please enter your age:
3. Are you of Hispanic, Latino, or Spanish origin?
  - No, not of Hispanic, Latino, or Spanish origin
  - Yes, Mexican, Mexican American, Chicano
  - Yes, Puerto Rican
  - Yes, Cuban
  - Yes, Central American (FILL IN):
  - Yes, South American (FILL IN):
  - Yes, Spanish (Spain)
4. Select the one group that best describes you.
  - White
  - Black/African American
  - Asian Indian
  - Chinese
  - Filipino
  - Japanese
  - Korean
  - Vietnamese
  - Other Asian (FILL IN):
  - Native American/American Indian/Alaskan Native (FILL IN Tribe):
  - Native Hawaiian or Other Pacific Islander
  - Mixed Ethnicity (example: Chicano and Native American, FILL IN):
  - Other (FILL IN):
5. Is English your first language?
  - Yes
  - No (please FILL IN your native language):
6. What is the highest level of education you have attained?
  - Did not finish high school
  - Graduated from high school
  - Attended some college but did not finish a 4-year degree
  - Graduated from a 4-year college or more
  - Obtained a graduate/professional degree
7. What is the highest level of education your father has attained?
  - He completed less than 12th grade (less than high school)
  - He graduated from high school
  - He had some college after high school
  - He graduated from a 4-year college or more
  - Don’t know
8. What is the highest level of education your mother has attained?
  - He completed less than 12th grade (less than high school)
  - He graduated from high school
  - He had some college after high school
  - He graduated from a 4-year college or more
  - Don’t know
9. How important would you say religion is to you?
  - Not at all important
  - Slightly important
  - Somewhat important
  - Important
  - Very important
10. What is your religious affiliation?
  - Catholic
  - Protestant (Methodist, Lutheran, Baptist, etc.)
  - Jewish
  - Evangelical/Born-Again Christian
  - Latter-Day Saint (Mormon)
  - Muslim
  - No religion
  - Other (FILL IN):
11. What is your current work status? You may select more than one. For example, if you are a part time student who also works part time, you should select part time student and working part time.
  - Working full time
  - Working part time
  - Retired
  - Disabled/unable to work
  - Unemployed, looking for work
  - Unemployed, not looking for work
  - Full time student
  - Part time student
12. When it comes to politics, would you describe yourself as liberal, conservative, or neither liberal nor conservative?
  - Very conservative
  - Conservative
  - Slightly conservative
  - Moderate; middle of the road
  - Slightly liberal
  - Liberal
  - Very liberal
13. Generally speaking, do you usually think of yourself as a Republican, a Democrat, an Independent, or something else?
  - Republican
    - Would you call yourself a strong Republican or not a very strong Republican?
      - Strong Republican
      - Not very strong Republican
  - Democrat
    - Would you call yourself a strong Democrat or not a very strong Democrat?
      - Strong Democrat
      - Not very strong Democrat
  - Something else
    14. Do you think of yourself as closer to the Republican Party or the Democratic Party?
      - Closer to the Republican Party
      - Closer to the Democratic Party
      - Neither
14. Do you approve or disapprove of the way Joe Biden is handling his job as President?
  - Strongly approve
  - Somewhat approve
  - Somewhat disapprove
  - Strongly disapprove
15. Generally, how interested are you in politics?
  - Extremely interested
  - Very interested
  - Somewhat interested
  - Not very interested
  - Not at all interested
